# Draft Genome Sequence of Bowmanella denitrificans JL63, a Bacterium Isolated from Whiteleg Shrimp (Litopenaeus vannamei) That Can Inhibit the Growth of Vibrio parahaemolyticus

**DOI:** 10.1128/genomeA.00215-18

**Published:** 2018-04-05

**Authors:** Jason P. LaPorte, Edward J. Spinard, Marta Gomez-Chiarri, David C. Rowley, John J. Mekalanos, David R. Nelson

**Affiliations:** a Department of Cell and Molecular Biology, University of Rhode Island, Kingston, Rhode Island, USA; b Department of Fisheries, Animal and Veterinary Sciences, University of Rhode Island, Kingston, Rhode Island, USA; c Department of Biomedical and Pharmaceutical Sciences, University of Rhode Island, Kingston, Rhode Island, USA; d Department of Microbiology and Immunobiology, Harvard Medical School, Boston, Massachusetts, USA

## Abstract

Bowmanella denitrificans strain JL63 was isolated from a whiteleg shrimp (Litopenaeus vannamei) and was determined to have antibacterial activity against an acute hepatopancreatic necrosis disease (AHPND) strain of Vibrio parahaemolyticus. Here, we report the draft genome sequence of this strain and identify genes that are potentially involved in its antibacterial activity.

## GENOME ANNOUNCEMENT

Bowmanella, a genus in the family *Alteromonadaceae* within the *Gammaproteobacteria*, was first identified in 2006 (1). Currently, only three species belonging to this genus have been described, Bowmanella denitrificans, Bowmanella pacifica, and Bowmanella dokdonensis. B. denitrificans is a chemoorganotrophic bacterium capable of respiratory, but not fermentative, metabolism (1). B. denitrificans strain BD1^T^, the first strain of this species to be identified, is capable of anaerobic growth by carrying out denitrification, whereas B. denitrificans strain S088 has been shown to produce a potent heat-stable algicidal compound (1, 2). B. denitrificans JL63 was isolated from a whiteleg shrimp (Litopenaeus vannamei). A zone-of-inhibition assay (3) determined that B. denitrificans JL63 can inhibit the growth of an acute hepatopancreatic necrosis disease (AHPND) strain of Vibrio parahaemolyticus on an agar surface. The B. denitrificans JL63 genome sequence reported here is the first draft genome sequence of a Bowmanella species.

B. denitrificans JL63 was grown overnight in yeast-tryptone broth supplemented with 3% artificial sea salts (LB30IOS) at 27°C. Genomic DNA was isolated using the Promega Wizard genomic DNA purification kit, and DNA was resuspended in 2 mM Tris-HCl buffer (Bio Basic). Sequencing was performed at the Rhode Island Genomics and Sequencing Center using an Illumina MiSeq sequencer. Sequence trimming was performed using CLC Genomics Workbench (version 9.5.3), resulting in 2,641,396 paired-end reads averaging 180 bp in size. Contigs with a coverage of ≥34× were assembled using the SPAdes genomic assembler (version 3.1.1) (4). The resulting contigs were processed using the CLC Microbial Genome Finishing module. The draft genome consists of 39 contigs, with a total sequence length of 5,479,914 bp and a G+C content of 50.4%. Gene annotation was performed using the Rapid Annotations using Subsystems Technology (RAST) server and resulted in 4,980 open reading frames (5–7). The 16S rRNA gene of B. denitrificans JL63 is 99.8% similar to that of B. denitrificans BD1^T^, 99.0% similar to that of B. pacifica W3-3A, and 95.0% similar to that of B. dokdonensis UDC354. The B. denitrificans JL63 *gyrB* and *rpoD* genes are 98.6% and 98.3% similar to those of B. denitrificans BD1^T^ and 81.2% and 80.8% similar to those of B. pacifica W3-3A, respectively.

The genome of B. denitrificans JL63 encodes several gene clusters that are potentially involved in the production of the following antibacterial compounds: lanthionine, colicin V, a secreted hemolysin-type calcium-binding bacteriocin, and the broad-spectrum antibacterial protein marinocine (encoded by the *lodAB* operon). The genome also encodes an antibiotic biosynthesis monooxygenase, a type VI secretion system (T6SS), and two iron acquisition systems (hemin and TonB), including the full complement of proteins responsible for the formation of the TonB-ExbB-ExbD complex. Gene clusters for a type IV pilus and a mannose-sensitive hemagglutinin type IV pilus system are also present. The genome contains gene clusters for denitrification and for nitrate/nitrite ammonification. Three secondary metabolic gene clusters in the JL63 genome were predicted using antiSMASH (8). Gene clusters predicted to synthesize a nonribosomal peptide and two bacteriocins, including lantipeptide, were identified.

### Accession number(s).

This whole-genome shotgun project has been deposited in DDBJ/ENA/GenBank under the accession no. PEBU00000000. The version described in this paper is the first version, PEBU01000000.
